# Subcutaneous white adipocytes express a light sensitive signaling pathway mediated via a melanopsin/TRPC channel axis

**DOI:** 10.1038/s41598-017-16689-4

**Published:** 2017-11-27

**Authors:** Katarina Ondrusova, Mohammad Fatehi, Amy Barr, Zofia Czarnecka, Wentong Long, Kunimasa Suzuki, Scott Campbell, Koenraad Philippaert, Matthew Hubert, Edward Tredget, Peter Kwan, Nicolas Touret, Martin Wabitsch, Kevin Y. Lee, Peter E. Light

**Affiliations:** 1 Alberta Diabetes Institute, Department of Pharmacology, Faculty of Medicine and Dentistry, University of Alberta, Edmonton, Alberta T6G 2E1 Canada; 2 Division of Plastic Surgery, Department of Surgery, Faculty of Medicine and Dentistry, University of Alberta, Edmonton Alberta, T6G 2B7 Canada; 3 Department of Biochemistry, Faculty of Medicine and Dentistry, University of Alberta, Edmonton, Alberta T6G 2E1 Canada; 4 Division of Pediatric Endocrinology and Diabetes, Interdisciplinary Obesity Unit, Department of Pediatrics and Adolescent Medicine, University of Ulm, Eythstr, 24/89075 Ulm, Germany; 5 Department of Biomedical Sciences, Heritage College of Osteopathic Medicine, Ohio University, Athens, OH 45701 USA

## Abstract

Subcutaneous white adipose tissue (scWAT) is the major fat depot in humans and is a central player in regulating whole body metabolism. Skin exposure to UV wavelengths from sunlight is required for Vitamin D synthesis and pigmentation, although it is plausible that longer visible wavelengths that penetrate the skin may regulate scWAT function. In this regard, we discovered a novel blue light-sensitive current in human scWAT that is mediated by melanopsin coupled to transient receptor potential canonical cation channels. This pathway is activated at physiological intensities of light that penetrate the skin on a sunny day. Daily exposure of differentiated adipocytes to blue light resulted in decreased lipid droplet size, increased basal lipolytic rate and alterations in adiponectin and leptin secretion. Our results suggest that scWAT function may be directly under the influence of ambient sunlight exposure and may have important implications for our current understanding of adipocyte biology. (150 words)

## Introduction

Dysfunctional white adipose tissue (WAT) is associated with the development of obesity, diabetes and cardiovascular disease^1^. The negative outcomes associated with WAT are mainly attributable to intra-abdominal visceral WAT^2,3^). However, subcutaneous WAT (scWAT), which comprises > 80% of the body’s WAT mass, also plays an active role in these diseases^4^. Therefore, an improved understanding of the regulation of scWAT function is of importance. Due to the sub-dermal localization of scWAT adipocytes over a large surface area of the human body, these cells may be directly affected by ambient sunlight exposure.

Humans are able to utilize ultraviolet light (<400 nm) for pigmentation and the biosynthesis of vitamin D^5–7^. In contrast, visible light (400–700 nm) has not garnered as much attention regarding extra-retinal effects, despite the fact that 38.9% of the sun’s energy reaching exposed skin is in the visible wavelength range^8^. Although the majority of sunlight in the visible spectrum does not pass through the skin, 1–5% of blue/green light is able to penetrate through human skin to varying depths proportional to increasing wavelength and intensity and reach the underlying scWAT^9^. Indeed, blue light has been recently shown to mediate subcutaneous vasorelaxation via activation of the blue light-sensitive *OPN4* gene product melanopsin^10^, a non-visual opsin best characterized in intrinsically photo-sensitive retinal ganglion cells^11–13^. Interestingly, high *OPN4* mRNA levels are also reported in human scWAT, but not visceral WAT (www.gtexportal.org/home/gene/OPN4). As a G_q_-protein-coupled receptor^14^, melanopsin acts via the activation of phospholipase C (PLC) and the production of inositol triphosphate (IP_3_), diacylglycerol (DAG) and subsequent activation of transient receptor potential canonical (TRPC) channels^13,15^, which mediate an influx of Ca^2+^ and Na^+^ ions^16,17^. Moreover, TRPC channels have also been shown to be present in pre-adipocytes and adipocytes^18–20^. Here we report on our serendipitous discovery and identification of a novel melanopsin/TRPC signaling pathway in scWAT adipocytes.

## Results

### scWAT adipocytes possess physiologically relevant blue light-sensitive inward currents

**Figure 1:**
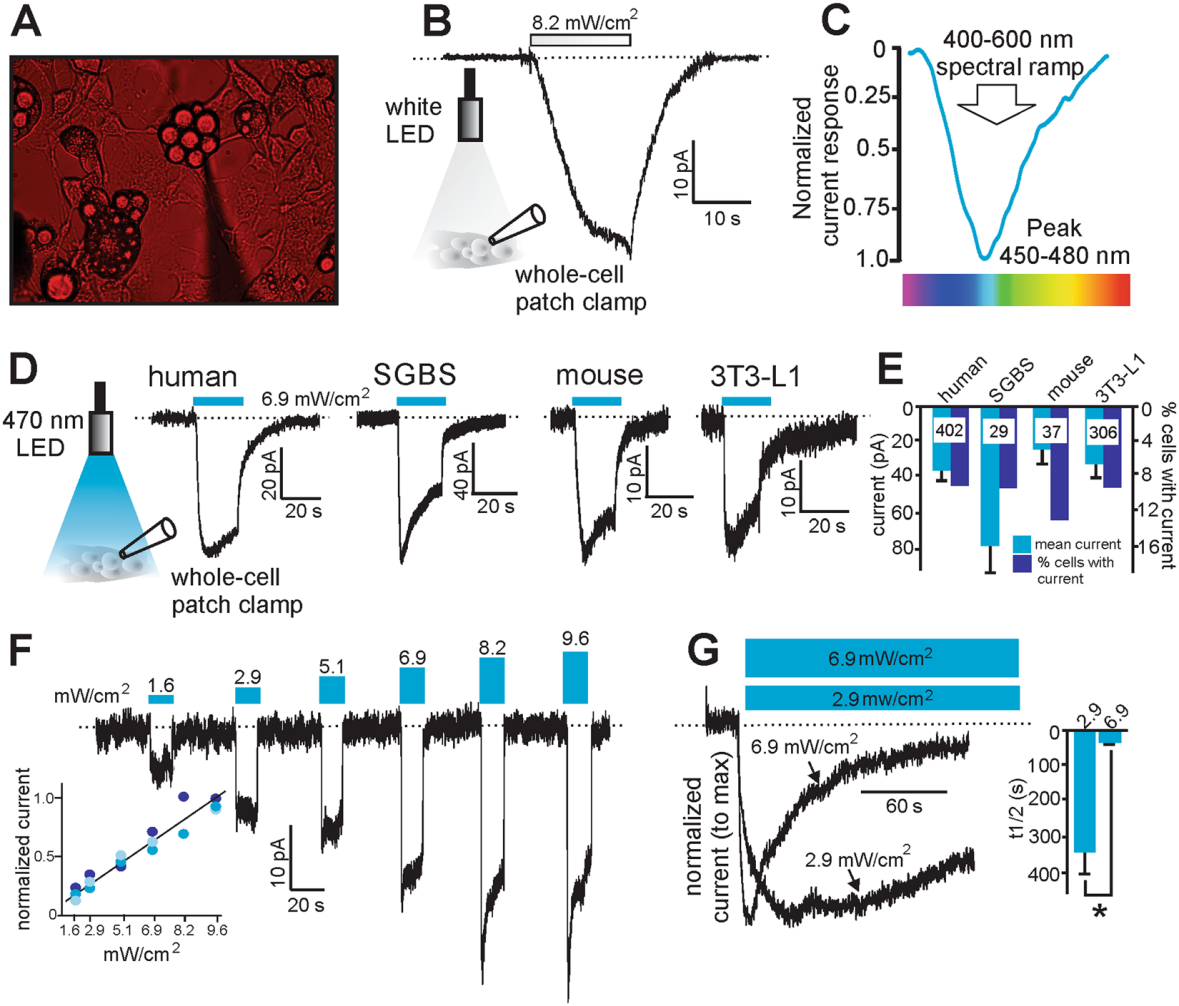
Differentiated adipocytes express a light-sensitive inward current. (**A**) Positioning of the electrophysiological recording pipette for whole-cell patch clamp recordings from adipocytes was made under red light conditions to prevent inactivation of any light-sensitive currents. (**B**) Representative recording of a light-sensitive inward current from a 3T3-L1 adipocyte in response to white light stimulation at an intensity of 8.2 mW/cm^2^. (**C**) Maximal currents were obtained at between 450–480 nm wavelength in 3T3-L1 adipocytes. (**D**) Representative recordings of blue light-sensitive inward currents in differentiated adipocytes from (1) human primary tissue (2) human SGBS cells (3) mouse primary tissue (4) mouse 3T3-L1 cells. Cells were stimulated with 470 nm blue light at an intensity of 6.9 mW/cm^2^. (**E**) Grouped data showing mean current obtained and the % of cells tested with measurable current. Numbers inside bars are the number of cells recorded from each group. (**F**) Representative recordings of the light-sensitive currents to 470 nm light at different intensities in 3T3-L1 adipocytes (inset = normalized currents from 3 cells at the tested light powers). (**G**) Representative recordings from 3T3-L1 adipocytes of the inactivation properties of these light-sensitive currents at two different light intensities of longer duration. Inset: time to inactivate to half maximal current was 26 +/− 3 s (n = 7) and 332 +/− 69 s (n = 3) at 6.9 and 2.9 mW/cm^2^ respectively. **Denotes P < 0.01. Statistical significance was determined using a Student’s t test. Dashed lines denote zero current level.

As bright white light is typically used in electrophysiology and many bi-stable opsins can inactivate under broad-spectrum light^21,22^, adipocytes were kept under red light to visualize the positioning of the recording pipette (Fig. 1A). Under these conditions, we observed a white light-induced inward current in cultured mouse 3T3-L1 differentiated adipocytes (Fig. 1B). To determine the wavelength sensitivity of this current, 3T3-L1 differentiated adipocytes were exposed to a 400–600 nm spectral ramp and the maximal current was elicited at ~450–480 nm blue light (Fig. 1C). Blue (470 nm) light-sensitive currents were observed in differentiated adipocytes from 1) primary human scWAT 2) SGBS cells (human pre-adipocyte cell strain^23^) 3) primary mouse inguinal scWAT, and mouse 3T3-L1 cells (Fig. 1D,E). In all of these cell types, ~10–13% of cells tested yielded measurable currents ranging between 35–80 pA in magnitude (Fig. 1E). Given the extensive previous validation of the 3T3-L1 cell line as a model of adipocytes^24^, we used these cells as our model system for the majority of our biophysical and functional characterization of this pathway. To characterize the current properties in response to different intensities of blue light, 3T3-L1 differentiated adipocytes were exposed to increasing light intensity: (1.6, 2.9, 5.1, 6.9, 8.2 and 9.6 mW/cm^2^). Current amplitude was proportional to light power with higher intensities inducing larger amplitude currents that exhibited more rapid inactivation (Fig. 1F). To investigate this further, cells were subjected to an extended 150 s exposure of  470 nm light at two different light intensities. Our results indicate the light-sensitive currents exhibit greater stability with significantly slower inactivation at the lower intensity (time to 50% reduction from peak current = 332 ± 69 s and 26 ± 3 s for 2.9 or 6.9 mW/cm^2^ respectively, Fig. 1G).

### The blue light-induced current in adipocytes is mediated through a melanopsin/ TRPC channel pathway

**Figure 2:**
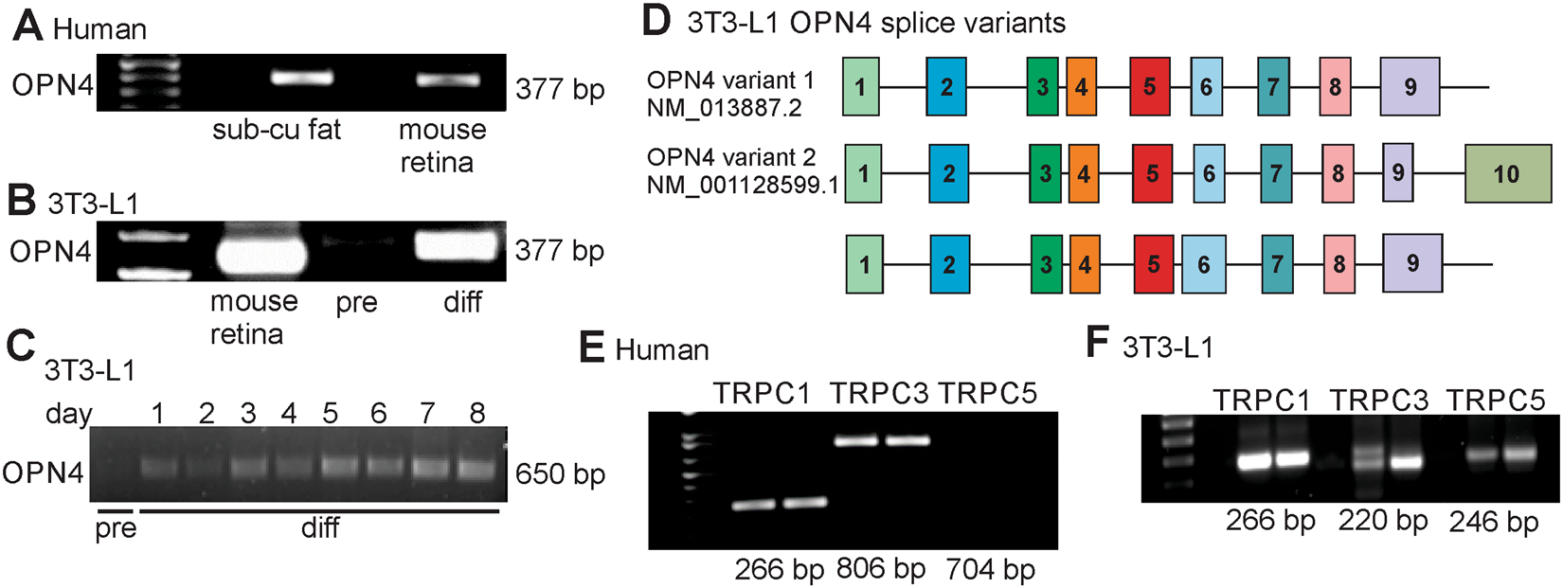
PCR analysis shows the existence of *OPN4* variants and TRPC channels in differentiated adipocytes. (**A**) Human scWAT shows message for *OPN4* (mouse retinal mRNA used as positive control). (**B**,**C**) *OPN4* message is present in 3T3-L1 differentiated (diff) adipocytes but not detectable in undifferentiated 3T3-L1 pre-adipocytes (pre). (**D**) Nested PCR analysis of exons 3 to 9, identified at least two *OPN4* splice variants in 3T3-L1 differentiated adipocytes (numbered boxes = exon makeup). Variants 1 and 2 are identical to known retinal *OPN4* variants (NM_013877.2 and NM_00128599.1). (**E**) Human scWAT shows message for *TRPC1/3*. (**F**) 3T3-L1 differentiated adipocytes shows message for *TRPC1/3/5*.

**Figure 3:**
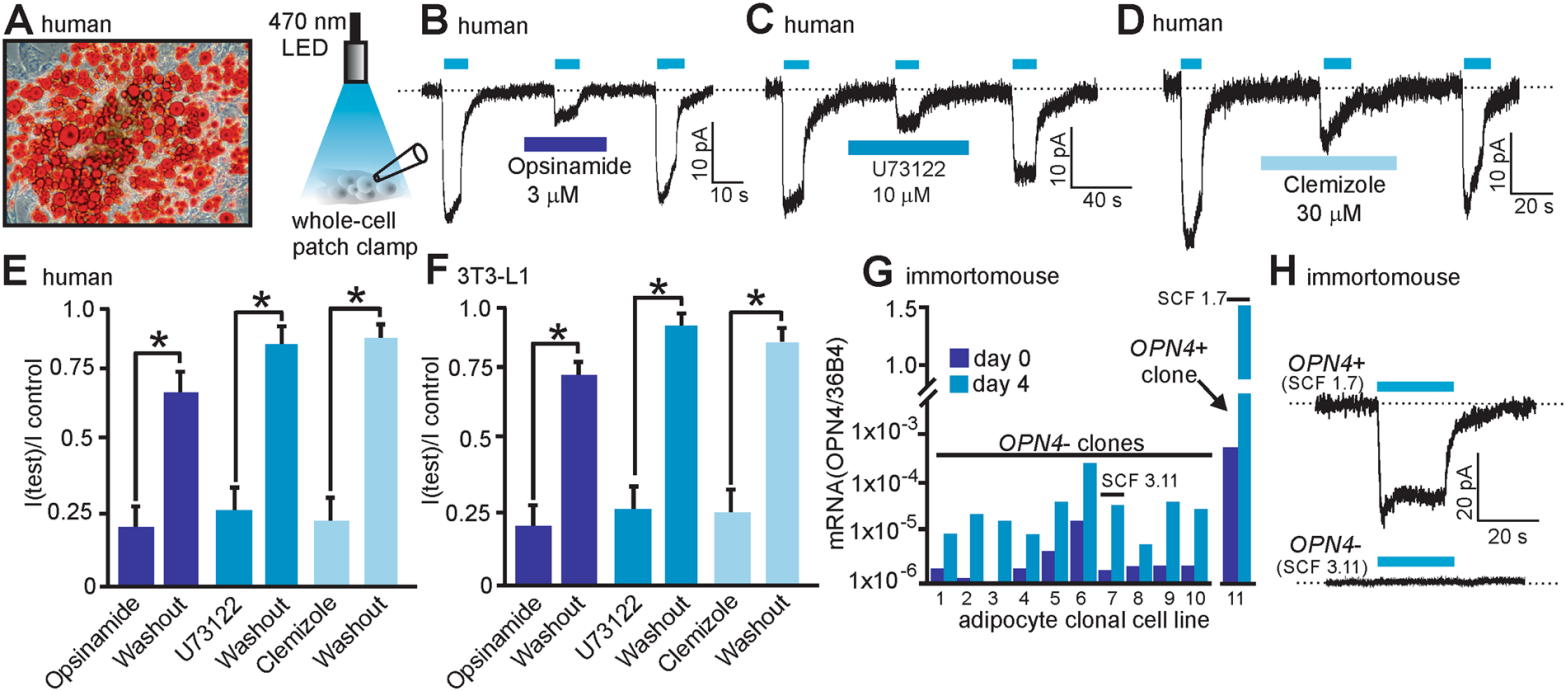
Pharmacological and molecular characterization of the light-sensitive current in differentiated adipocytes. (**A**) Human differentiated primary adipocytes stained with Oil Red-O for lipid content. (**B–D**) Representative whole-cell light-sensitive recordings from human primary adipocytes showing the inhibitory effects of the melanopsin inhibitor opsinamide (**B**), the phospholipase C inhibitor U73122 (**C**) and the TRPC channel inhibitor clemizole (**D**). (**E**,**F**) Grouped data from human primary adipocytes and 3T3-L1 adipocytes (n = 3–4 cells in human primary adipocytes and n = 4–6 cells for 3T3-L1 adipocytes). (**G**) Immortomouse clonal adipocyte cell line mRNA qPCR analysis reveals the presence of a single *OPN4* positive clone (SCF 1.7) from 11 clonal cell lines tested. Day 0 = day of differentiation and day 4 = 4 days post differentiation. *OPN4* message normalized to the 36B4 housekeeping message levels. (**H**) Only differentiated adipocytes from the *OPN4*+ clonal cell line (SCF 1.7) display light-sensitive currents. Light-sensitive currents were observed in 7 of 32 cells tested for the SCF 1.7 *OPN4*+ clonal line, whereas 0 of 23 cells tested displayed current in cells from the SCF 3.11 *OPN4*- clonal line. *Denotes P < 0.01. Statistical significance was determined using a paired one-tailed Student’s t test. (**B–D** & **H**) Blue bars above traces denote time of 470 nm light application at a power of 6.9 mW/cm^2^. Dashed lines denote zero current level.

As the maximal light-sensitive current elicited at ~450–480 nm blue light (Fig. 1C) is analogous to the spectral sensitivity of melanopsin^11,13,14^, we tested whether scWAT expresses mRNA transcripts for *OPN4* and *TRPC* channels. Using mouse retina as a positive control, we observed the presence of *OPN4* mRNA in human scWAT (Fig. 2A) and 3T3-L1 differentiated adipocytes, although *OPN4* message was not detected in undifferentiated 3T3-L1 pre-adipocytes (Fig. 2B). We confirmed this latter observation by measuring *OPN4* message in pre-adipocytes and at days 1–8 post-differentiation and we again did not detect *OPN4* message in pre-adipocytes until after differentiation (Fig. 2C). Additionally, nested PCR results indicate that two of the *OPN4* variants detected, between Exon 3 and Exon 9, are identical to the two major mouse retinal *OPN4* variants as well as detecting another *OPN4* variant with a larger Exon 6 (Fig. 2D).

Differentiated adipocytes were then analyzed for *TRPC1/3/5* mRNA as 1) melanopsin is thought to couple to TRPC channel isoforms^25^ 2) TRPC channels may form hetero-tetramers^26,27^ and 3) TRPC1 and 5 channels have been implicated in adipocyte function^20^. We observed *TRPC1/3* mRNA in adipocytes cultured from human scWAT (Fig. 2E) and *TRPC1/3/5* mRNA in 3T3-L1 differentiated adipocytes (Fig. 2F). To further validate the existence and properties of this putative melanopsin/TRPC signaling axis in scWAT, we used pharmacological inhibitors of melanopsin (opsinamide^28^), phospholipase C (U73122) and TRPC channels (clemizole^29^). In human scWAT differentiated adipocytes (Fig. 3A), the blue light-sensitive current was reversibly inhibited by the application of opsinamide (3 μM), U73122 (10 μM) or clemizole (30 μM) (Fig. 3B–E). Comparable results were obtained in 3T3-L1 differentiated adipocytes (Fig. 3F).

Interestingly, our results indicate that only 10–13% of cells tested exhibited measurable blue-light sensitive current (out of 774 cells in total patched from the 4 cell types used, Fig. 1E). These results suggest that the currents were either too small, already inactivated or that there are possibly distinct sub-populations of adipocytes that express this light-sensitive pathway. To test this latter concept and to provide further evidence of melanopsin-mediated light-sensitivity of adipocytes, we used qPCR to screen 11 immortalized clonal adipocyte cell lines for *OPN4* message (cells derived from scWAT from the immortomouse^TM^ model^30^). We identified one *OPN4* positive clone (SCF 1.7) out of the 11 clonal lines tested that was characterized by high *OPN4* expression upon differentiation (Fig. 3G). Importantly, we observed blue light-sensitive current only in *OPN4*+ SCF 1.7 differentiated adipocytes (7 of 32 cells tested, 22%) while no current was detected in differentiated adipocytes from the *OPN4−* SCF 3.11 clonal cell line (0 out of 23 cells tested, Fig. 3H).

### Daily blue light exposure affects lipid homeostasis and adipokine secretion in adipocytes

**Figure 4:**
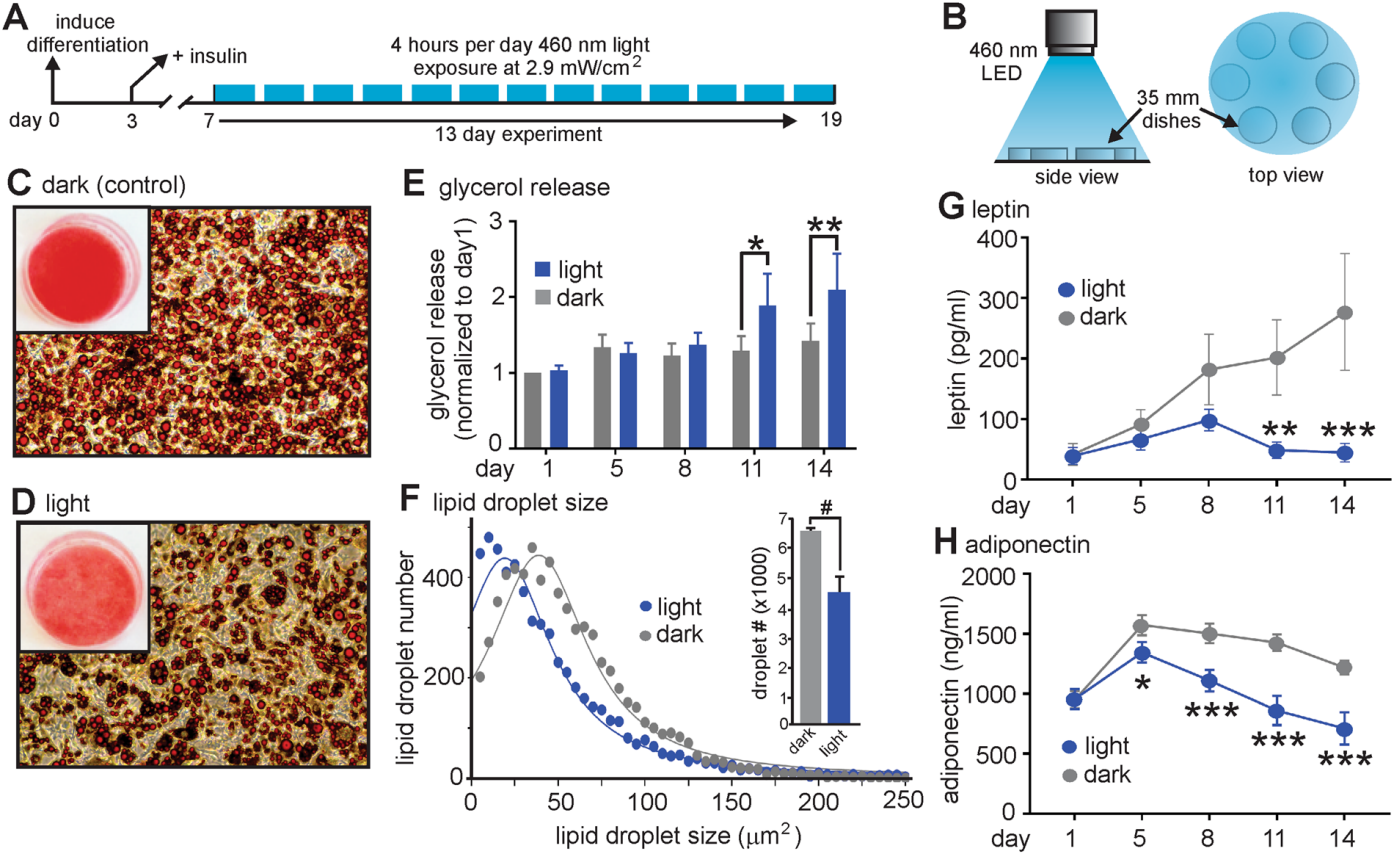
Chronic blue light treatment of 3T3-L1 differentiated adipocytes alters lipid storage and adipokine release. (**A**) Schematic illustration of the experimental protocol used for these experiments. (**B**) Experimental set-up for illumination of the differentiated adipocytes in 35 mm dishes. (**C**) Chronic exposure to blue light (460 nm) reduces Oil Red-O lipid staining when compared to non-light treated (dark) cells (**D**). Inset = Oil Red-O lipid staining in 35 mm culture dishes. (**E**) Glycerol release is significantly increased in light treated cells at days 11 and 14. (**F**) Lipid droplet size and number are significantly decreased in light-treated cells. Data were plotted as a frequency distribution with a bin size of 5 μm^2^. Since >99% of the lipid droplet sizes were at sizes lower than or equal to 250 μm^2^, data was plotted with a maximum size of 250 μm^2^ and fitted with a Lorentizan (least squares) nonlinear regression (n = 3 experiments per group). (**G**,**H**) Leptin and adiponectin secretion is significantly reduced in light-treated cells (n = 3 experiments per group). #, *,** and *** denote significant differences of P < 0.05, <0.01, 0.001, and 0.0001 respectively. Statistical significance was determined using either a paired one-tailed Student’s t-test (**F**) or a two-way repeated measures ANOVA followed by a Sidak multiple comparisons test (**G**,**H**).

To investigate whether chronic blue light exposure affects the phenotype and function of adipocytes, we exposed 3T3-L1 differentiated adipocytes to blue (460 nm) light at an intensity of 2.9 mW/cm^2^ that generates stable currents (Fig. 1F,G) for 4 hours daily over 13 consecutive days (Fig. 4A,B). Visual inspection of cells stained for lipid with Oil Red-O suggested reduced lipid content in the light-treated group compared to the control (dark) group (Fig. 4C,D). This observation may be explained by an increase in lipolysis that can be measured by assaying release of glycerol into the supernatant. Indeed, a significant increase in glycerol release at day 11 and day 14 was observed in the light treated group when compared to the control (dark) group (Fig. 4E). The presence of smaller lipid droplets would also indicate an increased basal rate of lipolysis. In this regard, quantification of lipid droplet size revealed that adipocytes in the light treated group contained significantly fewer lipid droplets compared to the control (dark) group (4758 ± 528 (light) vs 6558 ± 105 (dark), P < 0.05) that were also significantly smaller in size (median values = 23.3 ± 0.9 μm^2^ (light) vs 33.0 ± 1.6 μm^2^ (dark), P < 0.05), (Fig. 4F). Differentiated adipocytes also secrete adipokine hormones such as leptin and adiponectin^31,32^. We therefore investigated whether chronic blue light exposure alters the secretory profile of leptin and adiponectin. Blue light-exposed adipocytes secreted significantly less leptin at days 11 and 14 when compared to the control (dark) group (Fig. 4G). Adipocytes exposed to blue light also secreted lower amounts of adiponectin, with significant changes apparent starting at exposure day 5 (Fig. 4H). To test whether an acute exposure to blue light could elicit similar effects, adipocyte cultures were subjected to a 60 min blue light exposure. However, no significant changes in either adipokine release nor glycerol content in the media were observed (data not shown).

## Discussion

This paper reports a novel and entirely unexpected presence of a blue light-sensitive current in human scWAT that is mediated through a melanopsin/TRPC signaling pathway. It is perhaps unsurprising that this pathway has not been previously discovered as the amplitudes of the light-sensitive currents are very small and may be readily inactivated under the broad spectrum white light conditions commonly used in electrophysiology. Our findings also indicate that this melanopsin/TRPC pathway is present at low density in adipocytes for the following reasons. The unitary single channel conductance of TRPC1 and 3 is between 17–60 pS^16,17,33^. Assuming that a single TRPC channel is open 50% of the time when activated and generates a current of ~1–3 pA, a typical blue light-sensitive current of 50 pA indicates the presence of only ~30–100 TRPC channels being activated at the adipocyte cell surface. Although adipocytes are well documented as an endocrine cell type^31,32^, they do not behave like typical excitable endocrine cells such as pancreatic beta cells that generate action potentials to drive insulin secretion. Therefore, the small amplitude and stable nature of the light-induced current observed in adipocytes may generate a tonic basal influx of Ca^2+^ ions into the cell that regulates downstream cellular processes over a period of hours to days. With respect to signaling pathways potentially involved, activation of the G_q_-coupled melanopsin may signal through DAG-mediated PKC activation that is known to regulate adipocyte lipid metabolism^34^ and increase lipolysis^35,36^. In addition, intracellular Ca^2+^ signaling may occur through direct TRPC channel activation, G_q_-mediated IP_3_ production, and Ca^2+^ store release^16,17,37,38^. This may lead to activation of calcium-sensitive PKC isoforms as well as modulation of the calcineurin/NFAT signaling axis that is known to regulate genes involved in adipocyte metabolism and lipolysis^39^. If the melanopsin/TRPC channel axis regulates adipocyte gene transcription, then this may account for the requirement of chronic rather than acute blue-light exposure we observed in our study.

Our findings that blue light exposure of adipocytes causes increased glycerol release and reduced lipid droplet size imply a potential shift in lipid homeostasis toward increased rate of basal lipolysis or reduced fatty acid re-esterification. In this regard, human adipocyte size has been directly correlated with adipokine expression and secretion, whereby smaller adipocytes secrete lower levels of adipokines such as leptin and adiponectin^40^. Interestingly, we observed that chronic blue light-exposed adipocytes contain smaller lipid droplets and secrete less leptin and adiponectin compared to their control counterparts. Taken together, this suggests that the effects of chronic blue light exposure on adipokine secretion may be a downstream consequence of reduced adipocyte lipid content. Moreover, adipocyte size is known to be an integral factor determining WAT health with hypertrophied adipocytes being associated with insulin resistance and inflammation^41,42^. The chronic light-induced activation of the melanopsin/TRPC signaling pathway may therefore potentially serve as a protective mechanism.

Interestingly, the melanopsin/TRPC signaling pathway was observed in both human and murine scWAT. These findings in mice are potentially confounding as the nocturnal behavior, light impermeable fur, and scWAT depot localization do not suggest an obvious role for scWAT light-sensing. The presence of *OPN4* in scWAT in other species suggest a common ancestral origin that provided an evolutionary advantage that is conserved in many mammals with perhaps redundant, limited, or as of yet undetermined biological function. In contrast, scWAT in humans is predominately located just beneath the skin that has a total surface area of 1.5–2.0 m^2^ in the average human that may be exposed to sunlight. Furthermore, 38.9% of the sun’s energy at sea level is in the visible range^8^ with skin being exposed to 200–400 mW/cm^2^ of visible wavelength light on a sunny day. While UV light minimally penetrates the skin, longer visible wavelengths possess greater penetrance and may potentially regulate cellular function. Within the visible spectrum ~40% is in the blue/green wavelength range. Although the majority of this blue/green light is reflected, scattered or absorbed in the upper skin layers, 1–5% may reach the underlying scWAT^9^, representing ~1–8 mW/cm^2^ of light intensity that is much brighter than an artificially lit laboratory (0.2–0.4 mW/cm^2^, unpublished observations). Therefore, it is important to note that we observed stable light-sensitive currents within this same range of ~1–8 mW/cm^2^ suggesting that these currents are likely persistently activated by similar blue/green light intensities penetrating the skin on a sunny day.

Our results also demonstrate that the light-sensitive currents were only observed in 10–13% of cells tested. While this could be explained by extremely low expression of melanopsin/TRPC channels in some cells, limitations of the recording technique or by inactivation prior to recording, it may also suggest the presence of distinct sub-populations. In this regard, the presence of heterogeneous sub-populations of adipocytes within scWAT is an area of active study, and has the potential to reveal adipocytes with specialized functions^43–45^. Our observations in *OPN4*+ and *OPN4*- clonal cell lines partially supports this notion, as 22% (7 of 32) of *OPN4*+ cells exhibited current compared to 10–13% in the other cell types tested (Fig. 1E).

In summary, we have identified a novel light-sensitive signaling pathway in human scWAT that is sensitive to ambient light levels that penetrate the skin on a sunny day. As such, these findings may shed new light on our current understanding of adipocyte biology. It is clear that centrally mediated circadian rhythms play an important role in human health and our results provide initial information about how appropriate sunlight exposure to scWAT might act as a peripheral circadian sensor that contributes to metabolic health. In contrast, a lack of sufficient bodily exposure to sunlight may contribute to long-term scWAT dysfunction and the current epidemics of obesity, diabetes and cardiovascular disease.

## Methods

### Experimental models

#### Human scWAT

Tissue samples were obtained from subjects undergoing abdominoplasty surgery in accordance with institutional human ethics guidelines and informed consent was obtained from all subjects. Experimental procedures for the isolation of scWAT from human samples were approved by the University of Alberta Biosafety Office. The scWAT was excised from the overlaying skin tissue (up to 1 cm of scWAT depth from the skin was used). Non-fat tissue was removed, and the scWAT was then minced and rinsed several times. The scWAT was digested for 0.5 h at 37 °C in 1.5 mg/mL of collagenase type 1 (Worthington, Lakewood, NJ, USA) in KRH buffer, filtered through a 250 µm cell strainer and centrifuged at 1000 RPM for 3 min. The pre-adipocyte pellet was plated in DMEM culture media supplemented with 10% fetal bovine serum (FBS) and 1% penicillin/streptomycin (P/S). Two days post-confluency, differentiation was induced on differentiation day 0 by the addition of 172 nM human insulin, 1 µM dexamethasone, 0.5 mM isobutyl-methylxanthine, and 1 µM rosiglitazone supplemented with 10% FBS and 1% P/S. On differentiation day 3, the media was changed to media containing 172 nM human insulin, 10% FBS and 1% P/S. The cells were cultured in this media for the duration of their use as well as during experimental protocols.

#### 3T3-L1 adipocytes

3T3-L1 adipocytes were initially cultured as pre-adipocytes in DMEM supplemented with 10% bovine calf serum and 1% P/S. The differentiation protocol for 3T3-L1 cells was the same as for human primary adipocytes, except bovine insulin was used instead of human insulin.

#### Mouse scWAT

All animal study protocols for tissue isolation were approved by University of Alberta Animal Care and Use Committee (protocol #s AU00286 and AU01417). Inguinal scWAT was isolated from male C57BL/6 mice (ages 3–6 mo.). The scWAT was then digested for 75 min at 37 °C in 1 mg/mL of type 1 collagenase (Worthington, Lakewood, NJ, USA) in KRH buffer. Digested scWAT was filtered through a 250 µm cell strainer, centrifuged at 1500 RPM for 10 min, and the pre-adipocyte-containing pellet was re-suspended in DMEM with 10% FBS and 1% P/S and subsequently filtered through a 40 µm cell strainer. Pre-adipocytes were plated in DMEM with 10% FBS and 1% P/S and then differentiated using the same protocol for 3T3-L1 adipocytes.

#### SGBS adipocytes

The Simpson-Golabi-Behmel Syndrome (SGBS) human pre-adipocyte cell strain was provided by Dr. Martin Wabitsch (Ulm University, Germany) and cultured and differentiated as described previously (Wabitsch *et al*., 2001).

### Method details

#### Electrophysiology

The whole-cell patch-clamp technique was used to record currents from differentiated adipocytes. Prior to recording, adipocytes were incubated in an extracellular solution containing 1 µM all-trans retinal. All manipulations and visualizations of the recording chamber were conducted under low levels of red light to avoid inactivation of the current. Cells were superfused with bath solution containing (in mM) 140 NaCl, 5 CsCl, 1 MgCl_2_, 2 CaCl_2_, 10 glucose, 10 HEPES and 1 μM all-trans-retinal (pH 7.4).The pipette solution contained (in mM) 130 CsCl, 2 NaCl, 7 KCl, 0.2 Na-GTP, 1 MgCl_2_, 0.4 EGTA, 10 HEPES (pH 7.2). Cells were maintained at a holding potential of −50 mV. Blue (470 nm) light stimulation was delivered using an LED (M470F3, ThorLabs, Newton NJ, USA). Opsinamide (#509267, EMDMillipore, Etobicoke, ON, Canada), U73122 (#U6756, Sigma-Aldrich), and clemizole hydrochloride (#5371, Tocris, Avonmouth, UK) were all dissolved in DMSO prior to use. Light intensity was measured using X-Cite XR2100 Power meter (Excelitas Technologies, Mississauga, ON, Canada). A DeltaRam X monochromator was used to apply a spectral ramp of 400–600 nm wavelengths to cells (Photon Technology International, Edison NJ, USA). All recordings were performed using an Axon Instruments 200B patch-clamp amplifier, and Clampex 9.2 and Clampfit 9.2 software (Molecular Devices, Sunnyvale CA, USA) for data acquisition and analysis.

#### Reverse transcriptase PCR

Total RNA was isolated from all samples using TRIzol Reagent (ThermoFisher Scientific, Waltham, MA, USA) and then reverse transcribed using qScript cDNA SuperMix, (Quanta Biosciences, Beverly, MA, USA). Q5® High-Fidelity DNA Polymerase was used for RT-PCR and Phusion High-Fidelity DNA polymerase (NEB, Ipswich, MA, USA) was used for the nested PCR. PCR was performed with the cDNA reverse transcribed from 1 µg total RNA using the primer sets detailed in Table 1.

**Table 1:** Primer sets used for the PCR and nested PCR experiments detailed in Fig. 2.

Figure#, the primer was used	Gene	Genebank Accession#	Primer Name	Sequence	Location
Fig. 2A,B	OPN4	NM_033282.3 or NM_013887.2	Fm753 OPN4	5′GCCTACGTGCCCGAG3′	(858–872) or (812–826)
Fig. 2A,B	OPN4	NM_033282.3 or NM_013887.2	Rm1129 OPN4	5′ATGACGGCTGGCACC3′	(1239–1226) or (1188–1174)
Fig. 2C	OPN4	NM_013887.2	Exon4/5-Sense	5′GCCACCTTTCTTTGGTTGGAGTGCCTAC3′	(790–817)
Fig. 2C	OPN4	NM_013887.2	Exon8/9-Antisense	5′GTTTCTGTGTCTGTCCAGCCCACTTCAC3′	(1449–1422)
Fig. 2DFirst round of nested PCR	OPN4	NM_013887.2	Exon3/4-Sense	5′GGAGACAGGTTGCGAGTTCTATGCCTTC3′	Exon3/4 (598–625)
Fig. 2D First round of nested PCR	OPN4	NM_013887.2	Exon6/7-Antisense	5′GATGTGCGAGTATCCAGCAAAGGCCAC3′	Exon6/7 (1153–1127)
Fig. 2D Second round of nested PCR	OPN4	NM_013887.2	Exon5 Froward	5′TGCTGACATCCTGCTCCTG3′	Exon5 (831–849)
Fig. 2D second round of nested PCR	OPN4	NM_013887.2	Exon6 Reverse	5′TGACAATCAGTGCGACCTTGGC3′	Exon6 (1079–1058
Fig. 2E	TRPC1	NM_001251845.1	TRPC1-F	5′CATGGAGCATCATATTTCACATT3′	(1304–1326)
Fig. 2E	TRPC1	NM_001251845.1	TRPC1-R	5′GTTGTGAGCAACCACTTTGAG3′	(1550–1570)
Fig. 2E	TRPC3	NM_001130698.1	TRPC3-F	5′CTTCTCTCGGATTGCGTACATC3′	(1940–1961)
Fig. 2E	TRPC3	NM_001130698.1	TRPC3-R	5′GTTCATAACGAAGGCTGGAGATA3′	(2723–2745)
Fig. 2E	TRPC5	NM_012471.2	TRPC5-F	5′GA GAAGGGGGAC TATGCCAC3′	(1040–1059)
Fig. 2E	TRPC5	NM_012471.2	TRPC5-R	5′TGGTCATCTCGATGGTTGAG3′	(1724–1743)
Fig. 2F	TRPC1	NM_11643.3	TRPC1-F	5′CATGGAGCATCGTATTTCACATTCTTG3′	(1684–1713)
Fig. 2F	TRPC1	NM_11643.3	TRPC1-R	5′GTTGTGAGCCACCACTTTGAG3′	(1953–1933
Fig. 2F	TRPC3	NM_019510.2	TRPC3-F	5′GCCTTCATGTTCGGTGCTC3′	(450–468)
Fig. 2F	TRPC3	NM_019510.2	TRPC3-R	5′AGGTTCTCCTTCTTCAGCAGC3′	(670–650)
Fig. 2F	TRPC5	NM_009428.3	TRPC5-F	5′GCTTTCGATATGAAGTGCTTGACC3′	(2658–2681)
Fig. 2F	TRPC5	NM_009428.3	TRPC5-R	5′CTCGACGACTCGGATTTTGG3′	(2904–2885)

#### Nested PCR

As multiple PCR products were obtained by RT-PCR of *OPN4* cDNA due to the presence of multiple *OPN4* transcripts in 3T3-L1 differentiated adipocytes, nested PCR was performed to analyze *OPN4* transcripts between Exon3 and Exon7. A forward primer that spans Exon3 and Exon4, (5′GGAGACAGGTTGCGAGTTCTATGCCTTC3′) and a reverse primer that spans Exon6 and Exon7 (5′GATGTGCGAGTATCCAGC AAAGGCCAC3′) were used to amplify *OPN4* transcripts. The PCR fragments were then separated by a 0.8% agarose gel in TBE buffer. Two bands, approximately 550 bp and 900 bp, were excised and each PCR fragment was extraced from the gel using QIAquick Gel extraction kit (QIAGEN, Valencia, CA, USA). Second round of PCR was carried out using a PCR fragment as a template, an Exon5 forward primer (5′TGCTGACATCCTGCTCCTG3′) and an Exon6 reverse primer (5′TGACAATCAGTGCGACCTTGGC3′). The PCR fragments were separated by a 0.8% agarose gel and then a main PCR product of each template was extracted from the gel as described above. Nested PCR products were sequenced by The Applied Genomics Core, University of Alberta.

#### Chronic blue light exposure

Starting on differentiation day 7, 3T3 L1 differentiated adipocytes were exposed to a chronic blue light protocol for 4 h per day for 13 consecutive days. The cells were kept in DMEM media containing 172 nM bovine insulin, as outlined above for differentiated adipocytes. Every day prior to light stimulation, beginning with the first day, a 1 mL sample of the cell culture supernatant was taken. Blue light (460 nm) stimulation (2.9 mW/cm^2^ at bottom of culture dish) was delivered using a Solis460A LED (Thorlabs, Newton NJ, USA). The LED was positioned at a distance of 76 cm above a platform containing six 35 mm culture dishes arranged in a circle to ensure equal light intensity distribution (Fig. 4B). The stimulation protocol was as follows: light on for 1 h, 15 min break, light on for 1 h, 30 min break, light on for 1 h, 15 min break, light on for 1 h—for a total daily exposure time of 4 h.

#### Lipid and glycerol analysis

Oil Red-O lipid (ORO) staining was used to assess changes in lipid homeostasis on day 14 of the chronic light stimulation protocol after the last day of light exposure (day 13). Cells were fixed in Z-fix for 15 min at room temperature, and then washed in ddH_2_O. They were then incubated in 60% isopropanol for 5 min, followed by incubation in 3 parts ORO stock solution (in 99% isopropanol) and 2 parts ddH_2_O for 10 min, and then washed in ddH_2_O. ORO-stained adipocytes were imaged using the Evos XL core inverted microscope (ThermoFisher Scientific, Waltham, MA, USA). A glycerol assay kit (#MAK117, Sigma-Aldrich) was used to quantify glycerol release by analyzing 3T3-L1 cell culture supernatant samples from days 1, 5, 8, 11, and 14 of the chronic blue light treatment and control (dark) groups. The assay was used as per the manufacturer's instructions.

#### Adipokine secretion

Leptin and adiponectin levels were quantified in 3T3-L1 cell culture supernatant samples from days 1, 5, 8, 11, and 14 of the chronic blue light protocol using electrochemiluminescent assays (K15124C-1 and K152BXC-1, MesoScale Discovery, Rockville, MD, USA) as per the manufacturer’s instructions.

### Quantification and statistical analysis

Lipid droplet size analysis was performed using the Image Processing Toolbox in MatLab software (R2017a), with a custom written code to detect spherical, ORO-stained objects and obtain droplet area in pixels^2^ ^2^. Area values were then converted to µm^2^ using a manually determined conversion factor. Following image processing, GraphPad Prism 7.03 software was used to plot and analyze data as well as all statistical analysis (GraphPad Software, La Jolla, CA, USA). Details of the statistical analysis performed and definition of n numbers can be found in the figure legends. All values provided are mean ± S.E.M, with the exception of Fig. 4F where values are the means of the median values ± S.E.M.
